# 基于Eµ-TCL1转基因小鼠建立慢性淋巴细胞白血病小鼠模型

**DOI:** 10.3760/cma.j.cn121090-20241120-00461

**Published:** 2025-05

**Authors:** 曼姁 张, 沙 郭, 阿不都克力木 娜迪娅, 阿力木 谢仁古丽, 瑞 张, 雪娇 曾, 淋一 张, 冉冉 张, 建华 曲

**Affiliations:** 1 新疆医科大学第一附属医院血液病中心，乌鲁木齐 830054 Hematology Center, The First Affiliated Hospital of Xinjiang Medical University, Urumqi 830054, China; 2 新疆维吾尔自治区血液病研究所，乌鲁木齐 830001 Xinjiang Uygur Autonomous Region Hematology Research Institute, Urumqi 830001, China

**Keywords:** 白血病，淋巴细胞，慢性，B细胞, 模型，动物, CD5, CD19, Leukemia, lymphocytic, chronic, B-cell, Models, animal, CD5, CD19

## Abstract

**目的:**

利用免疫球蛋白重链增强子调控的T细胞白血病/淋巴瘤1（Eµ-TCL1）转基因小鼠脾细胞过继性转移（AT）至野生型（WT）小鼠体内，建立一种具有自身免疫功能、成模周期较短的慢性淋巴细胞白血病（CLL）小鼠模型。

**方法:**

实验使用了无特定病原体（SPF）级健康的C57BL/6J WT小鼠和H11-Eµ-VH-TCL1-β-globin-PolyA基因敲入小鼠。通过CRISPR/Cas9技术构建了H11-Eµ-VH-TCL1-β-globin-PolyA基因敲入小鼠，并采用PCR方法鉴定小鼠的基因型。实验动物被随机分为AT组和WT组，每组10只，AT组为H11-Eµ-VH-TCL1-β-globin-PolyA基因敲入小鼠脾细胞腹腔注射至体内的WT小鼠。监测小鼠的体重和一般情况，并在移植后第9周进行颈椎脱臼处死。通过病理学表现、外周血白细胞变化和免疫表型等关键指标对CLL小鼠模型进行疾病验证。

**结果:**

AT组脾重量为（0.92±0.16）g、肝重量为（2.11±0.56）g；WT组脾重量为（0.06±0.01）g、肝重量为（1.42±0.13）g，AT组出现明显的肝（*P*＝0.006）、脾（*P*<0.05）肿大。AT组外周血白细胞数量较WT组明显增多［（124.33±8.74）×10^9^/L对（5.55±1.67）×10^9^/L，*P*＝0.002］；AT组外周血B淋巴细胞百分比较WT组增多［（69.13±6.88）％对（39.78±5.94）％，*P*<0.05］。病理组织学检查发现AT组小鼠的脾、淋巴结、骨髓出现CLL病理表现，肝、肺、肾组织均有明显淋巴细胞浸润。流式细胞术检测结果示AT组CD19^+^CD5^+^ B淋巴细胞占淋巴细胞百分比在外周血、骨髓和脾中分别为（61.37±9.92）％、（28.61±7.08）％、（86.03±5.78）％；WT组CD19^+^CD5^+^ B淋巴细胞占淋巴细胞百分比在外周血、骨髓和脾中分别为（4.51±1.32）％、（5.58±1.46）％、（14.33±3.2）％；AT组外周血、骨髓、脾中CD19^+^CD5^+^ B淋巴细胞占比较WT组增多，差异均具有统计学意义（均*P*<0.05），且CD43、CD200表达阳性，但CD20、CD22、CD79b的表达低于WT组。

**结论:**

通过Eµ-TCL1转基因小鼠脾细胞AT，成功建立了成型周期相对较短的CLL小鼠模型，其可作为一种理想的临床前模型用于CLL相关疾病研究。

## Abstract (en)

**Objective:**

To generate a chronic lymphocytic leukemia（CLL）mouse model with intact immune competence and short latency by adoptively transferring（AT）splenocytes from immunoglobulin heavy-chain enhancer-driven T-cell leukemia/lymphoma 1（Eµ-TCL1）transgenic donors into wild-type（WT）recipients.

**Methods:**

Specific pathogen-free C57BL/6J WT mice and H11-Eµ-VH-TCL1-β-globin-PolyA knock-in mice were utilized. The H11-Eµ-VH-TCL1-β-globin-PolyA knock-in mice were generated using CRISPR/Cas9 technology, and their genotypes were confirmed by PCR. Experimental animals were randomly divided into an adoptive transfer（AT）group and a WT control group（*n*＝10 per group）. Mice in the AT group received an intraperitoneal injection of splenocytes from H11-Eµ-VH-TCL1-β-globin-PolyA knock-in mice. The weight and general condition of the mice were monitored. Mice were euthanized by cervical dislocation at 9 weeks post-transplantation. The CLL model was validated using key indicators, including pathological manifestations, changes in peripheral blood leukocyte counts, and immunophenotype.

**Results:**

AT group mice exhibited significantly increased spleen weight［（0.92±0.16）g *vs*（0.06±0.01）g in WT group, *P*<0.05］and liver weight［（2.11±0.56）g *vs*（1.42±0.13）g in WT group, *P*＝0.006］, indicative of marked splenomegaly and hepatomegaly. The peripheral blood leukocyte count was significantly higher in the AT group［（124.33±8.74）×10⁹/L］compared to the WT group［（5.55±1.67）×10⁹/L］（*P*＝0.002）. Similarly, the percentage of peripheral blood B lymphocytes was markedly increased in the AT group versus the WT group［（69.13±6.88）％ *vs*（39.78±5.94）％, *P*<0.05］. Histopathological examination revealed CLL manifestations in the spleen, lymph nodes, and bone marrow of AT group mice, with significant lymphocytic infiltration observed in the liver, lung, and kidney tissues. Flow cytometry analysis showed that the percentages of CD19^+^CD5^+^ B lymphocytes among total lymphocytes in peripheral blood, bone marrow, and spleen of the AT group were（61.37±9.92）％,（28.61± 7.08）％, and（86.03±5.78）％, respectively. These were significantly higher（all *P*<0.05）than in the WT group［（4.51±1.32）％,（5.58±1.46）％, and（14.33±3.20）％］. Furthermore, these CLL-like cells in the AT group were positive for CD43 and CD200, but showed lower expression of CD20, CD22, and CD79b compared to WT B cells.

**Conclusion:**

Adoptive transfer of splenocytes from Eµ-TCL1 transgenic mice successfully established a CLL mouse model with a relatively short latency period. This model represents a valuable preclinical tool for investigating CLL and related pathologies.

慢性淋巴细胞白血病（CLL）以成熟的CD5^+^ B细胞在血液、骨髓、脾和淋巴结中克隆性增殖并大量聚集为特征，通常发生在老年患者中[1]。目前关于CLL的发病机制尚未明确，付春玲等[2]和赵子璇等[3]利用人类CLL细胞株MEC-1建立了CLL小鼠模型，但其因免疫缺陷而无法进行CLL免疫机制的研究。因此，寻找一种具有自身免疫功能的CLL小鼠模型至关重要。免疫球蛋白重链增强子调控的T细胞白血病/淋巴瘤1（Eµ-TCL1）转基因小鼠模型是迄今为止唯一显示出淋巴结肿瘤浸润的CLL小鼠模型，并且在小鼠体内表现出强烈的CLL肿瘤细胞与T淋巴细胞的相互作用，可作为研究CLL免疫微环境的实验动物模型[4]。

T细胞白血病/淋巴瘤1（TCL1）基因位于14q32.1，其染色体的易位和倒位与T细胞慢性淋巴细胞白血病/T细胞幼淋巴细胞白血病（T-CLL/T-PLL）的发病相关[5]–[6]，TCL1蛋白是癌基因TCL1的产物，其作为AKT激酶的共激活剂，在CLL和淋巴瘤的发生发展中起着重要作用[7]。人TCL1基因在体内（Em-TCL1）的外源性表达在免疫球蛋白重链可变区（IGHV）启动子和IGH增强子的控制下可导致CD5^+^ B细胞的克隆扩增[8]，基于TCL1基因的过表达，Eµ-TCL1转基因小鼠可在衰老后（通常为13～18个月）发展为类似于人类的CLL[9]，然而该模型成型周期较长。本研究通过同种异体移植的方法，利用Eµ-TCL1转基因小鼠脾细胞过继性转移（AT）至野生型（WT）小鼠体内，建立具有自身免疫功能且成模周期较短的动物模型。

## 材料与方法

一、实验动物

无特定病原体（SPF）级的C57BL/6J WT小鼠购于新疆医科大学动物实验中心，H11-Eµ-VH-TCL1-β-globin-PolyA基因敲入小鼠购自江苏集萃药康生物科技股份有限公司。本实验共采用20只6～8周，体重为19～25 g的C57BL/6J小鼠（8只雌鼠、12只雄鼠），实验前随机将小鼠分为2组，每组10只，其中4只雌鼠、6只雄鼠。一组以C57BL/6J为遗传背景的WT小鼠为受体小鼠，移植Eµ-TCL1转基因小鼠的脾细胞为AT组；一组以C57BL/6J为遗传背景的WT小鼠为WT组。本实验所用小鼠均饲养于环境温度为20～24 °C、环境湿度为50％～70％的SPF级动物房内，保持正常日光周期、垫料干燥、自由进食及饮水。所有操作均符合新疆医科大学动物实验中心伦理要求（批件号：20210301-169）。

二、利用CRISPR/Cas9技术构建H11-Eµ-VH-TCL1-β-globin-PolyA基因敲入小鼠

H11-Eµ-VH-TCL1-β-globin-PolyA基因敲入小鼠的构建委托江苏集萃药康生物科技股份有限公司完成，构建过程简述如下：基于CRISPR/Cas9技术原理，利用显微注射技术构建相应的打靶载体，并根据TCL1基因制备对应的gRNA、同时构建同源重组供体载体（Donor vector），将CRISPR/Cas9系统和Donor vector样品显微注射到C57BL/6J背景的小鼠受精卵中，取注射后存活的受精卵移植到假孕雌鼠体内待其怀孕产仔；待受体小鼠生出的基因型为阳性的F0代小鼠性成熟后，与WT小鼠进行交配，得到聚合酶链反应（PCR）和测序鉴定阳性的F1代小鼠，基因敲入示意图见图1。基因型阳性F1代小鼠可用于保种建系，扩大种群，后代进行基因鉴定，基因型阳性者可作为供体小鼠。

**图1:**
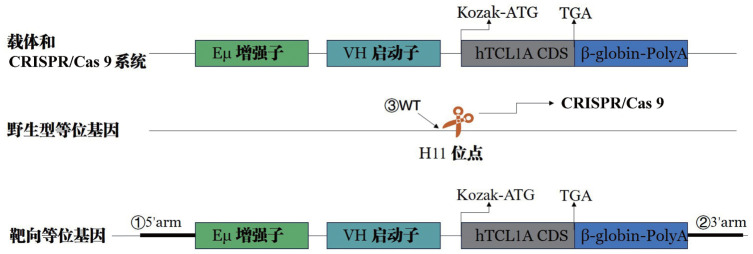
H11-Eµ-VH-TCL1-β-globin-PolyA小鼠基因敲入示意图 **注** Kozak-ATG：Kozak序列-ATG起始密码子；HTCL1A CDS：人源TCL1A基因编码序列；Β-globin-PolyA：β-珠蛋白-PolyA信号序列；TGA：终止密码子

三、PCR鉴定小鼠基因型

剪取出生1周小鼠尾尖0.5 cm置于1.5 ml的EP管中，加入500 µl消化液和50 µl蛋白酶K溶液（试剂均购于北京索莱宝科技有限公司），将上述EP管放入55 °C恒温水浴锅消化过夜，待消化完全，使用无水乙醇沉淀提取法提取基因组DNA。反应体系25 µl：2×Mix 12.5 µl，正反向引物［引物通过NCBI网站设计（NCBI号：8115），由上海生工生物工程股份有限公司合成］各1.0 µl，模板DNA 1.0 µl，ddH_2_O 9.5 µl；引物序列如下：引物1：5′arm：正向序列（F）1 TTCAGCAAAACCTGGCTGTGGA，反向序列（R）1 AGCCTGGACTTTCGGTTTGGT；引物扩增片段大小为681 bp；引物2：3′arm：F2 CGTGGAGGACATGCTTCTCG，R2 TCTCTATTG GCAGTTTGACACATCC；引物扩增片段大小为647 bp；引物3：WT：F3 AGTCTTTCCCTTGCCTC TGCT，R3 GGGTCTTCCACCTTTCT TCAG；引物扩增片段大小为825 bp；PCR反应程序：预变性95 °C，5 min；变性95 °C，30 s；退火58 °C，30 s；延伸72 °C，30 s/kb；变性到延伸共35个循环；最后再延伸72 °C，5 min；10 °C维持保存。PCR产物通过琼脂糖凝胶电泳进行鉴定。鉴定引物对应位点见图1。

四、Eµ-TCL1转基因小鼠AT模型的建立

以10月龄以上基因检测阳性的转基因小鼠为供体，取其脾，在两个磨砂载玻片之间的RPMI 1640培养基中分离组织、过滤。通过红细胞裂解液（北京索莱宝科技有限公司）裂解红细胞，然后在PBS中洗涤2次，细胞计数后将（2～4）×10^7^个脾细胞腹腔注射至6～8周性别对应的C57BL/6J WT小鼠体内。每周监测小鼠体重及一般情况，根据文献[10]及前期实验结果，在移植后第9周通过颈椎脱臼处死小鼠。

五、Eµ-TCL1转基因小鼠AT模型的鉴定

1. 病理表现：取每组小鼠脾、淋巴结、肝、肺、肾，观察其大小、颜色、质地等一般情况并称重。用4％多聚甲醛（北京索莱宝科技有限公司）浸泡小鼠各脏器过夜，固定后的组织进行组织脱水。组织切片后进行苏木精-伊红（HE）（北京索莱宝科技有限公司）染色，光镜下观察病理改变。

2. 外周血白细胞变化：通过眼球取血取小鼠外周血不少于60 µl置于EDTA-K2抗凝管，通过白细胞稀释液（购于北京索莱宝科技有限公司）进行白细胞计数，对比AT组和WT组小鼠外周血白细胞数量。10 µl用于制备外周血涂片，其余样本通过流式细胞术观察小鼠外周血淋巴细胞变化。

3. 免疫表型：通过流式细胞术检测小鼠外周血、脾、骨髓中CD5^+^CD19^+^ B淋巴细胞数量变化情况及外周血中CD23、CD43、CD200、CD20、CD22、CD79b（抗体PE anti-mouse CD5、FITC anti-mouse CD19、APC anti-mouse CD23、PE/Cyanine anti-mouse CD43、PE anti-mouse CD200、APC/Cyanine7 anti-mouse CD20、APC anti-mouse CD22、PE anti-mouse CD79b均购于BioLegend, Inc.公司，美国）的表达情况。

六、统计学处理：所有数据均至少进行3个生物学重复，实验数据通过SPSS 26.0整理分析，计量资料用*x*±*s*表示，两组间比较采用*t*检验，统计数据采用GraphPad prism 10.2进行绘图。以*P*<0.05为差异具有统计学意义。

## 结果

一、Eµ-TCL1转基因小鼠基因型鉴定

本实验中所用Eµ-TCL1转基因小鼠是以C57BL/6J为遗传背景的转基因小鼠，故本实验中以C57BL/6J为WT对照。WT小鼠样本在用引物3扩增后，可得一条825 bp的条带，而转基因小鼠样本则无对应条带。而利用引物1、2扩增后，转基因小鼠样本可分别得到一条681 bp和647 bp的条带，WT小鼠样本无对应条带，如图2所示，可知编号21、22、23、24、25、26、27的小鼠样本均含有目的基因。因此，本研究使用含目的基因的小鼠作为供体小鼠。

**图2:**
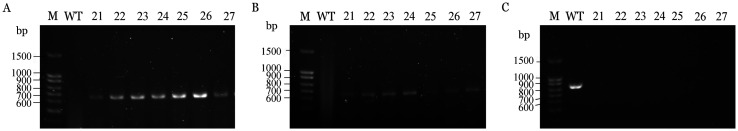
Eµ-TCL1转基因小鼠样本PCR产物琼脂糖凝胶电泳图 **A** 引物1扩增产物条带（681 bp）；**B** 引物2扩增产物条带（647 bp）；**C** 引物3扩增产物条带（825 bp） **注** Eµ-TCL1：免疫球蛋白重链增强子调控的T细胞白血病/淋巴瘤1；条带从左到右依次为Marker（M）、野生型（WT）、编号为21、22、23、24、25、26、27小鼠样本的PCR产物条带

二、移植后小鼠一般情况

在对WT小鼠进行脾细胞移植后，WT组与AT组小鼠体重都呈现增长趋势，体重变化差异无统计学意义（*P*>0.05）。移植后第7周，AT组1只雌鼠死亡，其余小鼠无死亡。移植后第9周，肉眼观察AT组2只雌鼠活力下降、嗜睡，其中1只体型明显缩小、体重减轻。AT组雄鼠与WT组无明显差异。

三、小鼠白血病表现

1. 病理表现：处死小鼠后可观察到：AT组脾重量为（0.92±0.16）g、肝重量为（2.11±0.56）g；WT组脾重量为（0.06±0.01）g、肝重量为（1.42±0.13）g，AT组出现明显的肝（*P*＝0.006）、脾（*P*<0.05）肿大；肺、肾重量差异均无统计学意义（均*P*>0.05）。HE染色结果显示：AT组可见脾小淋巴细胞形成大小基本一致的结节，红白髓边界不清；正常淋巴结结构被分化成熟的小淋巴细胞弥漫浸润，可见淡染的假滤泡；骨髓增生活跃，淋巴细胞显著增多，可见间质、结节或弥漫性浸润，以上提示AT组淋巴细胞浸润至肝、肺、肾，如图3、4所示。

**图3:**
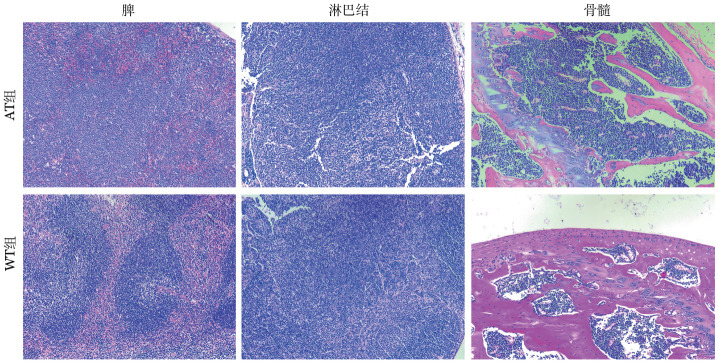
过继性转移（AT）组和野生型（WT）组小鼠脏器（×100，HE染色） **注** AT组：移植了免疫球蛋白重链增强子调控的T细胞白血病/淋巴瘤1（Eµ-TCL1）转基因小鼠脾细胞的以C57BL/6J为遗传背景的WT小鼠；WT组：未接受Eµ-TCL1转基因小鼠脾细胞移植的以C57BL/6J为遗传背景的WT小鼠

**图4:**
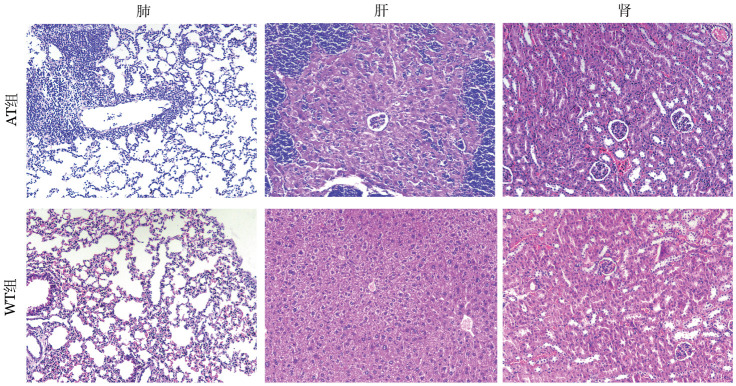
过继性转移（AT）组和野生型（WT）组小鼠脏器（×100，HE染色） **注** AT组：移植了免疫球蛋白重链增强子调控的T细胞白血病/淋巴瘤1（Eµ-TCL1）转基因小鼠脾细胞的以C57BL/6J为遗传背景的WT小鼠；WT组：未接受Eµ-TCL1转基因小鼠脾细胞移植的以C57BL/6J为遗传背景的WT小鼠

2. 外周血白细胞变化：AT组外周血白细胞计数较WT组明显增多［（124.33±8.74）×10^9^/L对（5.55±1.67）×10^9^/L，*P*＝0.002］。流式细胞术检测结果显示AT组外周血B淋巴细胞百分比较WT组增多［（69.13±6.88）％对（39.78±5.94）％，（*P*<0.05）］。显微镜观察外周血涂片显示，AT组小鼠较WT组外周血淋巴细胞增多，如图5所示。

**图5:**
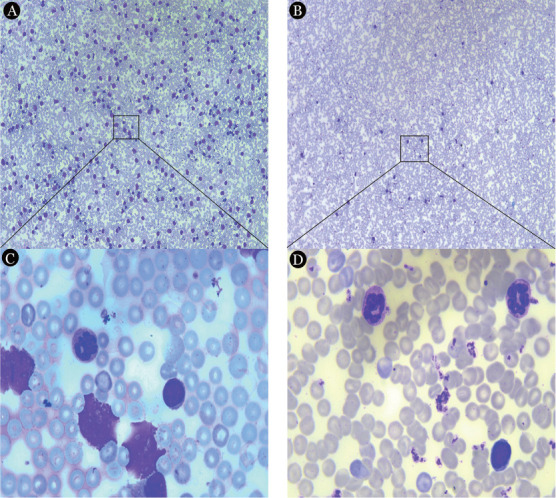
过继性转移（AT）组和野生型（WT）组外周血涂片 **A** AT组外周血涂片低倍（×100）；**B** WT组外周血涂片低倍（×100）；**C** AT组外周血涂片高倍（×1 000）；**D** WT组外周血涂片高倍（×1 000） **注** AT组：移植了免疫球蛋白重链增强子调控的T细胞白血病/淋巴瘤1（Eµ-TCL1）转基因小鼠脾细胞的以C57BL/6J为遗传背景的WT小鼠；WT组：未接受Eµ-TCL1转基因小鼠脾细胞移植的以C57BL/6J为遗传背景的WT小鼠

3. 免疫表型：流式细胞术结果显示：AT组CD19^+^CD5^+^ B淋巴细胞占淋巴细胞百分比在外周血、骨髓和脾中分别为（61.37±9.92）％、（28.61±7.08）％、（86.03±5.78）％；WT组CD19^+^CD5^+^ B淋巴细胞占淋巴细胞百分比在外周血、骨髓和脾中分别为（4.51±1.32）％、（5.58±1.46）％、（14.33±3.22）％；AT组外周血、骨髓、脾中CD19^+^CD5^+^B淋巴细胞占淋巴细胞百分比较WT组增多，差异均具有统计学意义（均*P*<0.05），结果如图6所示；AT组外周血CD43、CD200表达阳性，如图7所示；AT组CD20、CD22、CD79b在B细胞上的表达低于WT组，结果如图8所示。

**图6:**
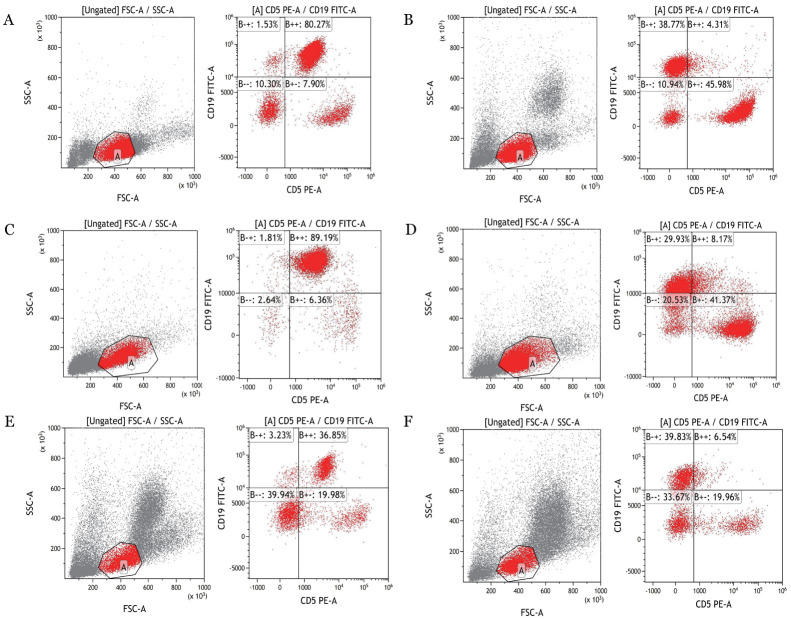
流式细胞术检测过继性转移（AT）组和野生型（WT）组CD19^+^CD5^+^ B淋巴细胞占淋巴细胞百分比 **A** AT组外周血CD19^+^CD5^+^ B淋巴细胞占上一门细胞百分比；**B** WT组外周血CD19^+^CD5^+^ B淋巴细胞占上一门细胞百分比；**C** AT组脾CD19^+^CD5^+^ B淋巴细胞占上一门细胞百分比；**D** WT组脾CD19^+^CD5^+^ B淋巴细胞占上一门细胞百分比；**E** AT组骨髓CD19^+^CD5^+^ B淋巴细胞占上一门细胞百分比；**F** WT组骨髓CD19^+^CD5^+^ B淋巴细胞占上一门细胞百分比 **注** AT组：移植了免疫球蛋白重链增强子调控的T细胞白血病/淋巴瘤1（Eµ-TCL1）转基因小鼠脾细胞的以C57BL/6J为遗传背景的WT小鼠；WT组：未接受Eµ-TCL1转基因小鼠脾细胞移植的以C57BL/6J为遗传背景的WT小鼠

**图7:**
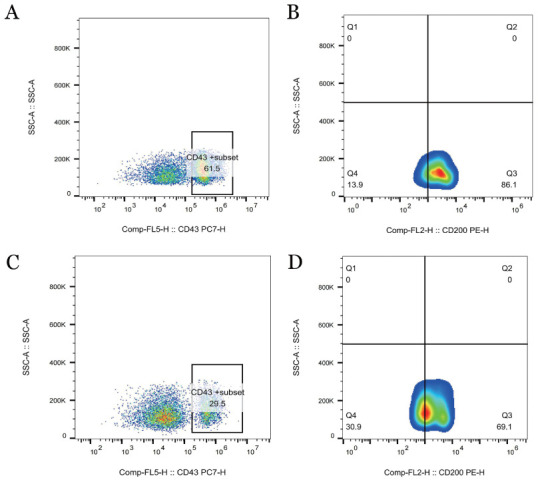
流式细胞术检测过继性转移（AT）组和野生型（WT）组外周血CD43和CD200的表达情况 **A** AT组外周血CD43表达情况；**B** AT组外周血CD200表达情况；**C** WT组外周血CD43表达情况；**D** WT组外周血CD200表达情况 **注** AT组：移植了免疫球蛋白重链增强子调控的T细胞白血病/淋巴瘤1（Eµ-TCL1）转基因小鼠脾细胞的以C57BL/6J为遗传背景的WT小鼠；WT组：未接受Eµ-TCL1转基因小鼠脾细胞移植的以C57BL/6J为遗传背景的WT小鼠

**图8:**
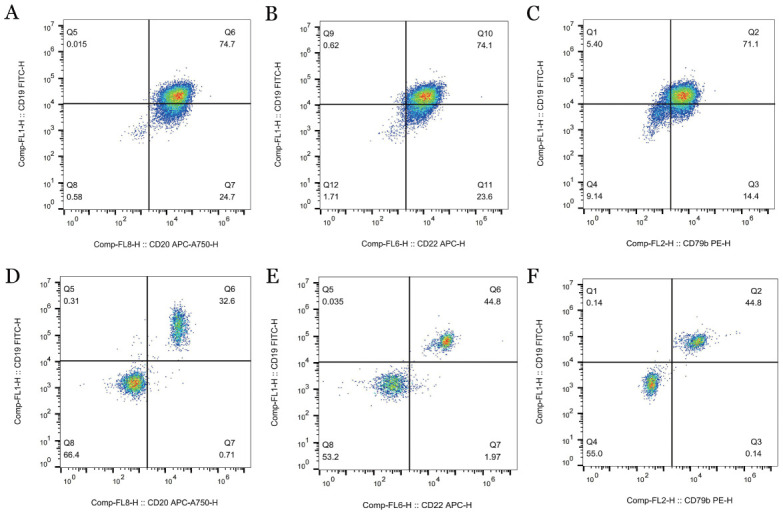
流式细胞术检测过继性转移（AT）组和野生型（WT）组外周血CD20、CD22和CD79b的表达情况 **A** AT组外周血CD20表达情况；**B** AT组外周血CD22表达情况；**C** AT组外周血CD79b表达情况；**D** WT组外周血CD20表达情况；**E** WT组外周血CD22表达情况；**F** WT组外周血CD79b表达情况 **注** AT组：移植了免疫球蛋白重链增强子调控的T细胞白血病/淋巴瘤1（Eµ-TCL1）转基因小鼠脾细胞的以C57BL/6J为遗传背景的野生型小鼠；WT组：未接受Eµ-TCL1转基因小鼠脾细胞移植的以C57BL/6J为遗传背景的野生型小鼠

## 讨论

本研究通过CRISPR/Cas9技术成功复刻了Eµ-TCL1转基因小鼠，经观察发现，该小鼠在7个月时，外周血涂片可见淋巴细胞增多；在13个月时，流式细胞术检测出现CD19^+^CD5^+^ B淋巴细胞比例明显增高，显微镜下观察外周血涂片淋巴细胞增多、白细胞计数增多，故该小鼠可在13个月以后自发进展为CLL，这与Bichi等[9]实验结果基本一致。

CLL淋巴细胞在体外几乎无自发增殖，且在离体培养时易发生细胞凋亡[11]。本实验中，Eµ-TCL1转基因小鼠脾细胞的体外培养并未获得扩大的细胞群，说明单纯免疫组织细胞共培养不能复现CLL淋巴细胞增殖所需微环境，Zanesi等[12]通过AT发现IgM^+^CD5^+^细胞能在小鼠体内蓄积，为本实验提供了理论基础。

CD5^+^ B淋巴细胞在外周血、骨髓和各免疫器官中进行恶性克隆性增殖是CLL的一个主要特征，CLL患者通常有体重减轻，肝、脾肿大等临床表现[13]。本研究解剖小鼠发现，AT组肝、脾均明显大于WT组；白细胞计数结果显示AT组外周血白细胞增多；外周血涂片发现AT组外周血成熟淋巴细胞增多；HE染色结果显示AT组小鼠脾小淋巴细胞形成大小基本一致的结节，红白髓边界不清，淋巴结中可见淡染的假滤泡、骨髓增生活跃，淋巴细胞显著增多以及出现淋巴细胞的非免疫器官浸润；流式细胞术显示AT组外周血、骨髓、脾中CD19^+^CD5^+^ B淋巴细胞大量蓄积。以上结果均表明，通过Eµ-TCL1转基因小鼠脾细胞AT方法构建的CLL小鼠模型已能够表现出CLL疾病的基本特征。

Eµ-TCL1转基因及AT组小鼠总体寿命情况：Eµ-TCL1转基因雌鼠中位生存期为367 d；AT组雌鼠中位生存期为73 d；Eµ-TCL1转基因雄鼠中位生存期为402 d，AT组雄鼠中位生存期为98 d。雌鼠寿命总体不及雄鼠长，Eµ-TCL1转基因小鼠患病时间通常为13～18个月，而构建的AT模型小鼠患病时间大大缩短（6～9周），且移植后受体小鼠外周血、脾和骨髓中CD19^+^CD5^+^ B淋巴细胞大量蓄积，蓄积程度大于供体小鼠，这可能提示，通过AT构建的CLL小鼠模型疾病程度高于Eµ-TCL1转基因小鼠且雌鼠疾病程度更为严重。有研究表明IGHV未突变的CLL患者疾病侵袭性更高[14]，而Eµ-TCL1转基因小鼠中B细胞受体显示出了其特征[15]，这提示Eµ-TCL1转基因小鼠疾病或更类似于人类侵袭性CLL；人类中含有MYC的染色体区域8q24的扩增与CLL患者的不良结局相关[16]，因此推测AT小鼠更为严重的疾病表现或与上述基因突变相关。为进一步研究该模型小鼠与人类CLL的差异，本实验完善了相关的免疫表型检测，结果发现，AT组3只雌鼠CD23表现为阴性、1只雄鼠CD23低表达，2只雌鼠CD20、CD22、CD79b表达水平明显高于WT组，CD43、CD200的表达情况基本符合人类CLL表达特点，后续实验中，将优化实验条件即在不存在需要立即采取行动的严重症状的情况下，在90％和100％的水平呈现疾病时对小鼠实施安乐死，期间对小鼠进行一般情况及疾病动态变化观察，并进一步探索转基因小鼠及其AT模型与人类CLL疾病进展的差异及机制。

通过异源性（如人源性CLL细胞）移植构造CLL小鼠模型周期较短（6～8周）[17]，但通常采用免疫缺陷鼠或实验前破坏小鼠自身免疫功能[2]–[3]，本研究采用鼠源性CLL细胞进行同种异体移植，为了避免发生免疫排斥反应（在本实验中免疫排斥反应可能会导致造模失败），采用与Eµ-TCL1转基因小鼠同遗传背景的C57BL/6J小鼠作为受体[18]，利用这种造模方式，CLL小鼠模型具有相对完整的免疫功能，且相对于Eµ-TCL1转基因小鼠，AT小鼠成模周期缩短，可提高实验效率，为CLL相关的免疫机制研究提供良好的临床前实验材料。

尽管Eµ-TCL1转基因小鼠及其AT模型保留了小鼠自身的免疫功能，但Eµ-TCL小鼠主要依赖TCL1癌基因过表达驱动B细胞恶性增殖[4]，而人类CLL的发病涉及多基因协同作用（如NOTCH1、SF3B1、TP53等高频突变）及染色体异常［如del（13q）、del（11q）］等[19]。有研究[20]表明实验室小鼠和人类在免疫细胞类型的相对丰度、基因表达模式以及特定基因功能方面存在显著差异。本研究缺乏对AT小鼠疾病的动态观察，在AT小鼠与Eµ-TCL1转基因小鼠发病及疾病表现差异方面未进行更深入的研究。因此，与人类CLL相比，本研究小鼠模型可能无法完全模拟复现CLL患者疾病进展中广泛存在的基因突变的累积、克隆演化过程以及微环境的重塑过程。

综上所述，利用Eµ-TCL1转基因小鼠脾细胞AT能在较短周期内成功制备CLL小鼠模型，为CLL发病、免疫及治疗等研究提供临床前模型。
